# Call for action: misinformation about Tourette syndrome from major media network

**DOI:** 10.1007/s00787-025-02939-8

**Published:** 2026-01-20

**Authors:** Kirsten R. Müller-Vahl, Natalia Szejko, Andreas Hartmann, Nanette Mol Debes, Tammy Hedderly, Mered Parnes, Elia Abi-Jaoude, Katarzyna Smilowska, Christelle Nilles, Keith Coffman, Andrea E. Cavanna, Julio Quezada, Claire Eccles, Cristiano Termine, Simon Schmitt, Catharina Cramer, Carolin Sophie Klages, Daniel Alvarez-Fischer, Susanne Munck Klansoe, Donald L. Gilbert

**Affiliations:** 1 Clinic of Psychiatry, Social Psychiatry and Psychotherapy, Hannover Medical School, Hannover, Germany; 2 Department of Bioethics, Department of Experimental and Clinical Pharmacology, Medical University of Warsaw, Warsaw, Poland; 3 Department of Neurology, National Reference Center for Tourette Syndrome, Pitié-Salpêtrière Hospital, Paris, France; 4 Department of Pediatrics and Adolescents Medicine, Copenhagen University Hospital - Herlev and Gentofte, Herlev, Denmark; 5 Department of Clinical Medicine, University of Copenhagen, Copenhagen, Denmark; 6 Department of Childrens Neurosciences Evelina London Childrens Hospital, London, UK; 7 Kings College London, London, UK; 8 Pediatric Movement Disorders Clinic, Section of Pediatric Neurology and Developmental Neuroscience, Texas Children’s Hospital and Baylor College of Medicine, Houston, USA; 9 Department of Psychiatry, Hospital for Sick Children, University of Toronto, Toronto, Canada; 10 Department of Psychiatry, Toronto Western Hospital, University of Toronto, Toronto, Canada; 11 Department of Neurology, 5th Regional Hospital, Sosnowiec, Poland; 12 Department of Neurology, Hôpital Fondation Rothschild, Paris, France; 13 Department of Neurology, Division of Child and Adolescent Neurology, Mayo Clinic, Rochester, MN USA; 14 Department of Neuropsychiatry, BSMHFT and University of Birmingham, Birmingham, UK; 15 Department of Child and Adolescent Neuropsychiatry, School of Medicine and Surgery, University of Milano-Bicocca, Milan, Italy; 16 Department of Neurodevelopmental Medicine, Kennedy Krieger Institute, Baltimore, MD USA; 17 The Tic Service, Great Ormond Street Hospital, Great Ormond Street, London, UK; 18 Department of Medicine and Innovation Technology, University of Insubria, Varese, Italy; 19 Institute of Systems Motor Science, University of Lübeck, Lübeck, Germany; 20 Division of Neurology, Cincinnati Children’s Hospital Medical Center and Department of Pediatrics, University of Cincinnati College of Medicine, Cincinnati, USA; 21 Department of Psychiatry, Psychotherapy and Psychosomatics, RWTH Aachen University, Aachen, Germany

“Stop that! It’s not Tourette’s but a new type of mass sociogenic illness” - this was the title of an article published in BRAIN in August 2021 [1] which received much public interest with a total of 108,647 views as of June 14th 2025. What prompted this commentary? Beginning in 2019, the incidence of adolescents and adults presenting for medical attention with impairing, dramatic, functional tic-like behaviors (FTLB) dramatically increased. As this increase reached global proportions during the COVID-19 pandemic and lockdowns, experts in Tourette Syndrome (TS) and FTLB realized this “outbreak” was a distinct clinical entity that could be confused with chronic tic disorders such as TS [2, 3]. FTLB is not TS, although some individuals may have both conditions [3]. FTLB is best conceptualized as a subtype of functional neurological disorder (FND). Subsequent actions by international academic collaborations, individual physicians, and advocacy groups contributed to a better clinical understanding of this disorder and formulation of diagnostic criteria [4]. Important factors that helped to raise awareness about this rapidly expanding clinical presentation of FTLB were international collaborations and efforts which provided physicians with assistance in making accurate diagnoses and providing appropriate treatments.

TS is defined as a chronic, childhood-onset disorder with combined motor and vocal tics lasting for at least a year [5, 6]. On average, tics start at the age of five to seven years, most typically with mild simple motor tics such as eye blinking and grimacing, followed by simple vocal tics such as sniffling and coughing, typically about two years later. Tics typically wax and wane and reach their maximum at age 10 to 12 years.

FND usually presents to clinicians as “disease mimics”. For example, individuals may manifest symptoms that resemble seizures, dissociative episodes, paralysis or weakness, tremor, or tics. Prior to 2019, FND presenting as TS was considered a very rare phenotype of FND that primarily presented with movements and sounds that overlapped more closely with standard clinical presentations of TS [7, 8]. Since 2019, however, individuals with FND mimicking TS presented much more commonly with predominantly complex movements and vocalizations such as obscene and socially unaccepted words, phrases and gestures, throwing objects, self-injurious behaviors, and long verbal phrases [9]. The term FTLB has come into wider use as a result of this phenomenon.

In 2023, an international group of Tourette experts, including many of the authors of this commentary, established diagnostic criteria for FTLB based on a Delphi consensus that outlined supportive factors for the diagnosis of FTLB and the differential diagnosis of TS [4]. They proposed that a clinically definite diagnosis of FTLB can be confirmed by an age at first symptom onset of 12 years or older, rapid onset and evolution of symptoms, and at least four of nine further phenomenological features as outlined in Table 2. Although there are clear “positive signs” for the diagnosis of FTLB, a better description of this patient group is needed to exclude circular reasoning due to a lack of clinical benchmarks [10].

Linked to the rise in FTLBs were two popular influencers, a young German man, who created the YouTube channel “Gewitter im Kopf” (eng. “Thunderstorm in the brain”) and a young English woman, who used the name “This Trippy Hippie” on TikTok. During the COVID-19 pandemic, these videos attracted tens of millions of views. In studies from several different countries and across continents, it could be clearly demonstrated that these presentations on social media directly influenced not only onset, but also clinical presentation of FTLB [1, 11–13]. In particular, according to an international database including 294 patients from ten tertiary referral centers for tic disorders from eight countries (Canada, United Kingdom, Germany, Australia, United States, Italy, Hungary, and France), FTLB was mainly seen in young female patients with mean age of 15 years (median 14 years; range, 8– 53 years), a demographic profile quite different from TS [2]. In fact, there is about a 4:1 predominance ratio of the male sex in TS, while FND, including FTLB, is mainly seen in females. Furthermore, an overlap could be demonstrated in the type of movements (mainly complex arm and hand movements, head banging and tapping) and vocalizations (often with a large number of swear words, whistles, insults, and comments) as well as socially inappropriate behaviors (including spilling drinks, hitting, and throwing food and objects) between social media influencers and patients [14]. Even more, depending on the place of residence and language of the patient, differences between clinical pattern and sex distribution were found, suggesting that exposure to particular social media content influences clinical presentation [1, 15, 16]. In this context, it is also important to mention that, while such behaviors are completely atypical for TS, mass use of social media easily creates misconceptions about TS [17] (Table 1).

**Table 1:** Examples of difference between features typical for TS and common false image of TS in media

Characteristic feature	TS	False image of TS in media
Age of onset	5–7 years	Any age
Onset and course of tics	Gradual	Sudden
Main tic phenomenology, especially at the beginning	Simple motor (e.g., eye movements, neck movements) and vocal tics (e.g., sniffing, throat clearing)	Complex movements (e.g., self-injury, hitting others) and sounds (e.g., coprolalia, repetition of phrases)
Coprophenomena	Very rare, present in only around 5% of patients and later in disease course	Frequent and almost pathognomonic for TS
Tics are context specific	No	Yes
Presentation of disease as being dangerous to the individual and the surrounding	Very rare	Could be present (e.g. self-injury, hitting others, destroying objects)
Impact of disease on quality of life	Mainly minor	Severe

*TS* Tourette syndrome

Importantly, among the general public at present, there are many misconceptions about what TS is. According to a large representative survey done in Germany in 2024, nearly half of participants stated that they were aware of TS. Symptoms that were classified as particularly characteristic of TS were “swear words and insults” followed by “obscene gestures and socially inappropriate behaviors” and “loud screams” (unpublished data). However, TS, in contrast to these now widely held beliefs, is mainly characterized by simple motor tics with short and abrupt movements and simple vocal tics such as coughing and sniffling [18]. In fact, coprolalia, copropraxia or disinhibited behaviors are found only in a minority of patients with TS. In the cohort of Nilles et al. [18] coprolalia was found in only 5% of cases, copropraxia in 3%, and paroxysms of tics in 7%.

It is important to understand what could be the possible reasons for how inaccurate views arose. It is our contention that, despite fewer patients with FTLB presenting clinically in the past year, there have been persistent misperceptions about the nature of TS generated by inaccurate media content. A potential large contribution to this public misperception has been social media influencers portraying themselves as having TS while presenting with videos of dramatic behaviors and phrases. For example, most adults with TS perform tasks adequately, if not exceptionally, in their adult lives. This is because tics typically decrease during the performance of an important motor action. It is therefore possible, for example, to play a musical instrument perfectly, to excel in sports, and even to perform a medical surgery with agility despite having tics. This contrasts markedly with the social media portrayal of supposed “tics” that lead people to perform uncontrollable, exaggerated actions such as tossing up eggs or throwing flour during cooking and lashing out at others. Such misrepresentations not only lead to stigmatization of those affected, but also may create great concern and fear in a parent of a child newly diagnosed with TS (Table 2).

**Table 2:**
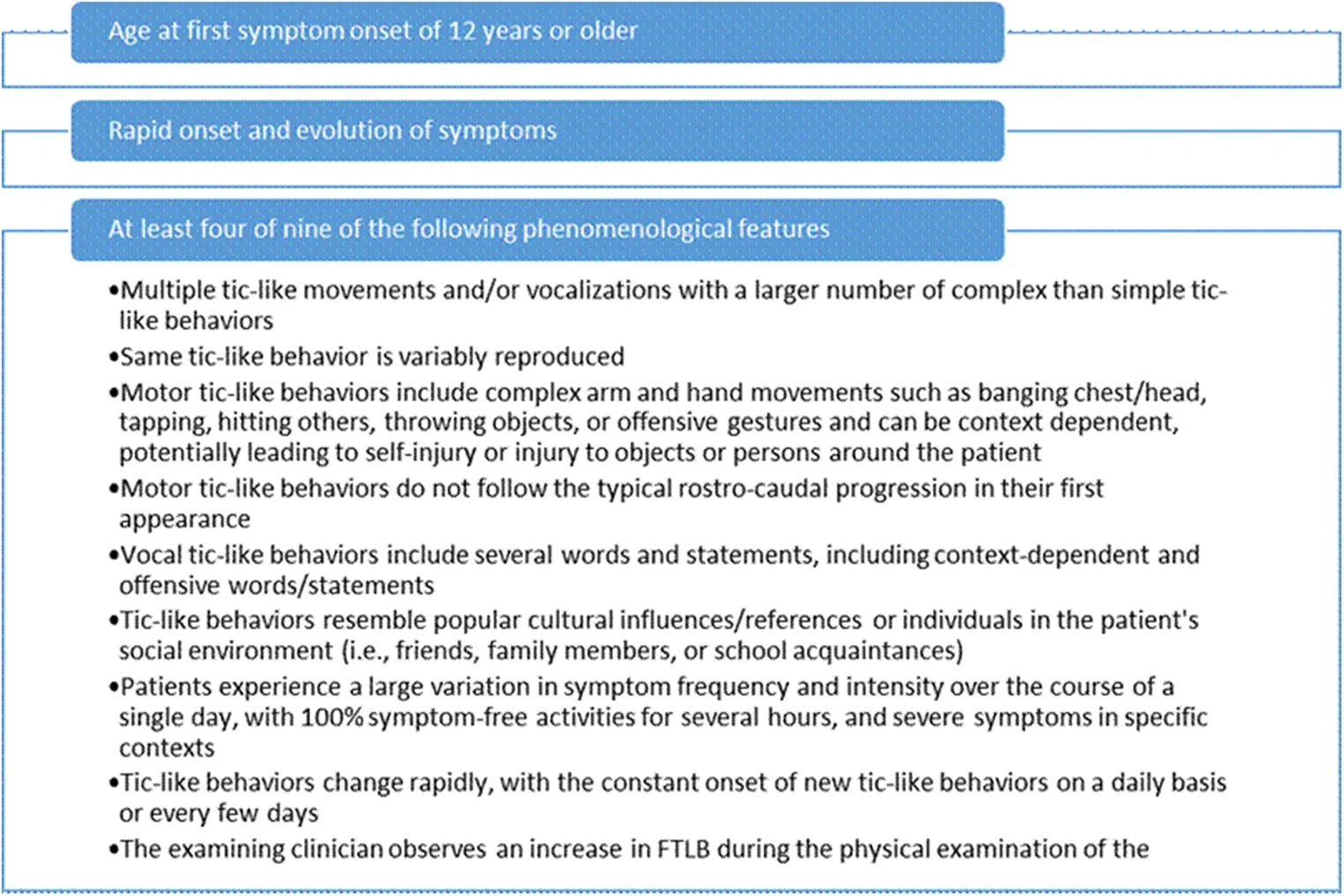
Clinically definite diagnostic criteria for FTLB

With the emergence of “Baylen Out Loud” on The Learning Channel which “follows the journey of a young woman living with an extreme case of TS”, and its support by the United States-based Tourette Association of America (TAA), we clinicians as well as patients and their families find themselves at a risk of more misunderstanding about TS and, possibly, a resurgence of FTLB. Accordingly, we are concerned about another step backwards that might further contribute to propagation of misconceptions regarding the TS.

Fundamentally, social media and media in general, including newspapers, blogs, TV, streaming programs, and films are in an ideal position to help raise awareness of the differences between FTLB and TS, reducing risks of harm from misdiagnosis. There are numerous examples of the important and positive role of the media in raising awareness or, for example, in helping young people to connect to other young people with FTLB. However, in a recently published study by Stamelou et al. [19], 66% of Youtube videos claiming to show movement disorders were rated by a group of international movement disorders experts as more typical for functional movement disorders. In another study [20], it has been shown that FND are often mistakenly shown as other disorders or “mystery disease”.

Traditionally, patient advocacy groups have also played a vital role in educating the public and correcting misperceptions of TS. An important example of this occurred in June 2019, when the German Tourette advocacy group (Tourette Gesellschaft Deutschland, TGD e.V.) publicly distanced themselves from the YouTube channel “Gewitter im Kopf”since they believed that this type of presentation and its content jeopardize their work aiming to improve education and public relations work about TS. Furthermore, they expressed their conviction that this channel had negative effects on TS awareness at school, at work or in public and led to increased stigmatization and exclusion [21]. Similarly, in July 2022, the UK patient organization Tourettes Action published a press release where they informed that behaviors and views shown in the documentary “Britain’s Tourette Mystery” launched by BBC Radio 4 [22] are not typical for TS [23]. In this show not only were the complex movements and vocalizations more typical for FTLB, they were sometimes labelled as typical for tics. Fortunately, the show also included an interview by one of our authors to provide medical information. Furthermore, in many countries and continents, several articles have been published in the lay press and documentary reports have been created informing about TS and FTLB, and the differences between these conditions. Finally, TS experts from the University in Calgary in Canada provided accurate information on FTLB on their website. Possibly partly as a result of these many efforts to provide medically accurate information, the incidence of extreme presentations of FTLB decreased greatly.

Previously, the TAA in the United States played a similar role to these other organizations. For example, in 2021, members of the Medical Advisory Board of the TAA, including several authors of this commentary, launched a comprehensive effort to inform the public about the differences between FTLB and tics. A detailed educational statement was posted entitled “Rising Incidence of Functional Tic-Like Behaviors” [24].

We therefore were surprised and became concerned that the TAA currently supports and promotes a reality TV show named “Baylen Out Loud” [25]. Under the heading “Baylen Out Loud – Learn More About Tourette Syndrome”, the TAA states that it is “grateful for Baylen’s efforts to raise awareness by bravely sharing her experience with TS, coprolalia, and other associated conditions.” However, based on more than three years of videos available on YouTube, TikTok, and other sites, the majority of symptoms seen on the show as well as on many videos online prior to the show, are phenomenologically more consistent with FTLB than TS [6]. In our view, the TAA’s support of the show helps to spread misleading information, further hindering correct diagnoses and classification of both TS and FTLB.

Following the Goldwater rule [26] according to which exact diagnosis cannot be established only on the basis of the videos or observation of the person without appropriate anamnesis and in person examination, the authors want to highlight that we do not want to distract from this important point by discussing diagnoses of individuals portrayed on television or social media. In addition, we are absolutely aware that people can have both TS and FTLB [3, 8], as this is also well-known for other neurological conditions. We therefore cannot - and also do not want to - exclude the additional diagnosis of TS in this case. Instead, we want to raise attention to ethics and responsibility of advocacy groups, television shows, social media, and scientific organizations to provide accurate medical information. This commentary does not violate the Goldwater rule, since Baylen Dupree herself makes it public that she suffers from an illness. Our primary goal is to draw attention to the fact that promotion of the TV show “Baylen out load” has the potential to mislead. We hereby also seek to alert other media groups to some potential harms that happen with misinformation.

We must stress that differentiating primary tic disorders such as TS from FTLB is not just an academic exercise, but it serves multiple vital purposes and principles for patients, their families, and clinicians:

1. We can explain to patients and families that FTLB (like other FND) are often managed differently and can have different prognosis, to adult TS;
2. It can prevent misuse of antipsychotics, motor targeted medications and other inappropriate treatments within the management of FTLB;
3. It allows access to helpful psychological treatments such as cognitive behavioral therapy (CBT);
4. For those who present with both FTLB and TS, it allows clinicians to better target tic-based therapies and medications appropriately, as necessary;
5. It prevents invalidation of patients with TS affected by severe symptoms through being told that their symptoms “are not as bad as a media person`s”;
6. It may help the social media influencers, whose symptoms might well be sustained, subconsciously, by relative fame and monetary gains, something termed recently “social media associated abnormal illness behavior” [27];
7. It helps correct a persisting caricatural representation of TS in the public, evolving around coprophenomena and extreme disinhibited behavior. Deepening understanding and awareness for TS does not, in our opinion, require dramatic representations but reasonable and measured ones.

## Conclusion

The portrayal of TS in many forms of media as a disorder primarily characterized by involuntary, complex movements, swearing, obscene gestures, and extended vocalizations is misleading. Inaccurate portrayals, particularly when targeted to young people, have potential to cause harm. We therefore wish to alert clinicians about a potential resurgence in FTLB, which happened before in Germany and the UK [1] and to raise awareness of the combined occurrence of tics/TS and FTLB. We further ask advocacy groups, and in particular the TAA as a well-known and renowned advocacy group with much influence in and outside the US, to stop promoting “Baylen Out Loud”. Instead, we advocate playing a positive role by informing the public about both diagnoses, TS and FTLB, and their differences. In addition, we encourage to include a scientific board of experts when selecting content spread on the websites and social media. While it is crucial that content selected by advocacy groups should be patient-oriented, it should also be scientifically accurate. Similarly, policymakers in health organizations should develop media campaigns in collaboration with a group of experts. Finally, the authors of this paper would be happy to consult or contribute to future educational material incorporating these values.

## Data Availability

No datasets were generated or analysed during the current study.
